# Periodic GFN2‑*x*TB in CP2K: Multipolar Ewald Electrostatics, k‑Point Sampling, and Transferability Benchmarks for Solids

**DOI:** 10.1021/acs.jctc.6c01034

**Published:** 2026-09-08

**Authors:** Vahideh Alizadeh, Johann Pototschnig, Leopold M. Seidler, Sebastian Ehlert, Thomas D. Kühne

**Affiliations:** † Center for Advanced Systems Understanding (CASUS), Conrad-Schiedt-Straße 20, Görlitz 02826, Germany; ‡ Helmholtz-Zentrum Dresden-Rossendorf, Bautzner Landstraße 400, Dresden 01328, Germany; § Mulliken Center for Theoretical Chemistry, Universität Bonn, Beringstraße 4, Bonn 53115, Germany; ∥ AI for Science, 214606 Microsoft Research, Karl-Liebknecht-Str. 32, Berlin 10178, Germany; ⊥ Institute of Artificial Intelligence, Technische Universität Dresden, Helmholtzstraße 10, Dresden 01069, Germany

## Abstract

Extended tight-binding methods are attractive for condensed-phase simulations because they retain an explicit electronic Hamiltonian at a cost far below conventional density functional theory. Their transferability to periodic materials, however, cannot be assessed reliably without a genuinely periodic Hamiltonian, Brillouin-zone sampling, and symmetry-aware crystal calculations. We present a periodic implementation of the second-generation geometry, frequency, and noncovalent interaction parametrization of extended tight binding (GFN2-*x*TB) in CP2K. The central theoretical ingredient is a multipolar Ewald formulation for the self-consistent shell charges, atomic dipoles, and quadrupoles of GFN2-*x*TB, together with analytical forces and stress contributions for geometry optimization, cell optimization, and molecular dynamics. For the ranking of ice polymorphs in the DMC-ICE13 data set, periodic GFN2-*x*TB on a converged 3^3^ *k*-point mesh improves the relative polymorph energies over GFN1-*x*TB, reducing the mean absolute error from 8.01 to 3.46 kJ mol^–1^. Because GFN1-*x*TB and GFN2-*x*TB differ in several coupled, jointly parametrized terms, this whole-model comparison does not isolate the contribution of multipolar electrostatics. For the LC10 set of cubic covalent and ionic solids, GFN2-xTB yields valid equation-of-state minima for all ten systems, with lattice-constant and cohesive-energy mean absolute errors of 0.062 Å and 1.30 eV per atom, respectively. Periodic GFN2-*x*TB is evaluated for molecular crystal lattice energies and cell parameters using the X23b data set. Using native Bloch 2^3^-mesh cell optimization with full symmetry reduction, followed by a 3^3^-mesh energy evaluation on the optimized cells, periodic GFN2-*x*TB gives a mean absolute lattice-energy error of 14.09 kJ mol^–1^ and a mean absolute relaxed-cell volume error of 5.84% for the full X23b relaxed-cell set. The benchmarks position periodic GFN2-*x*TB as an efficient electronic structure level for screening, pre-optimization, and large exploratory simulations. This work creates the foundation for periodic anisotropic electrostatics in the extended tight-binding framework and their efficient application in CP2K.

## Graphical abstract

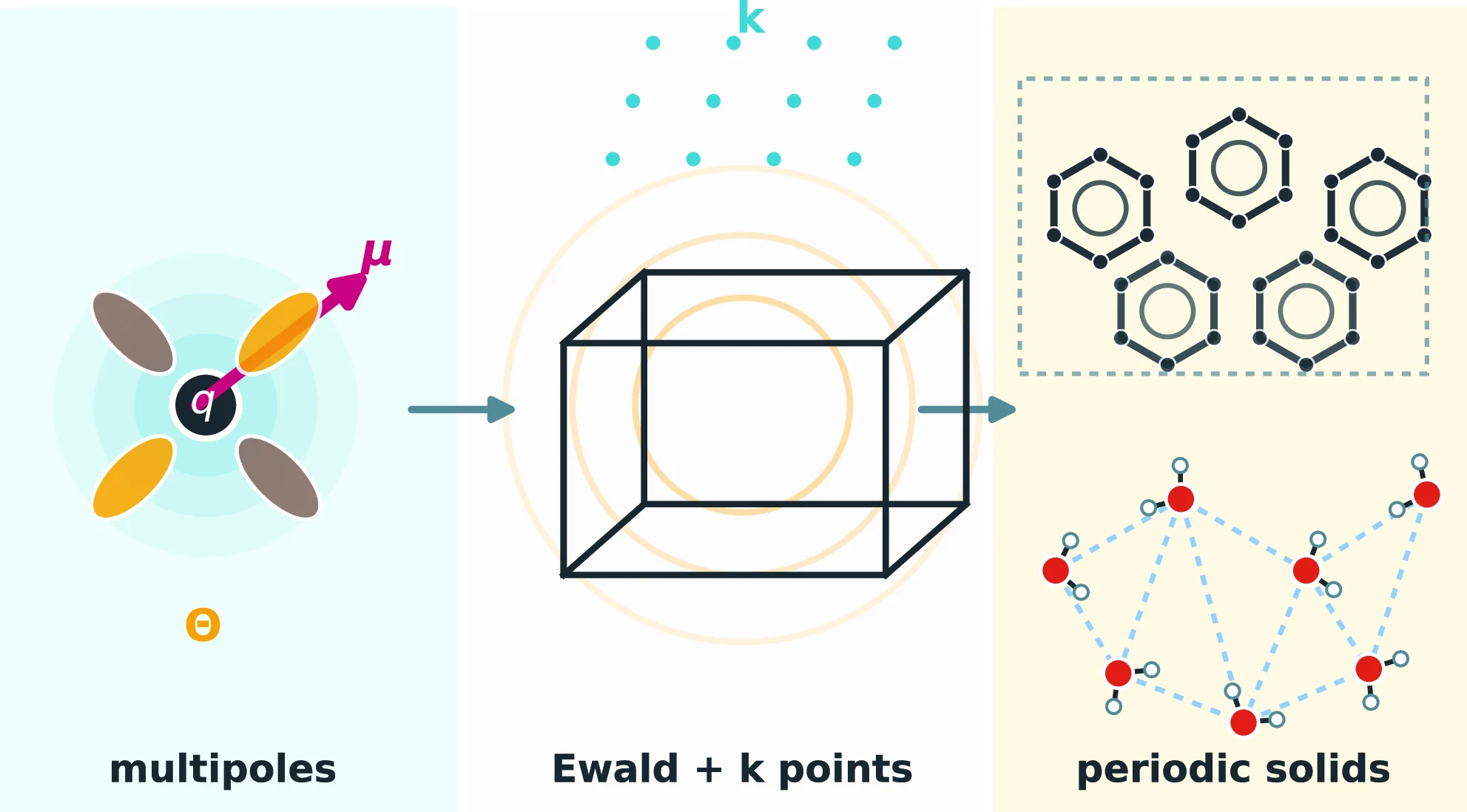

## 1 Introduction

Semiempirical quantum chemical (SQC) methods occupy an important middle ground between transferable classical force fields and first-principles electronic structure theory. They retain an explicit electronic Hamiltonian and therefore provide access to charges, orbitals, bond orders, response properties, and bond breaking, but they replace the most expensive electronic structure ingredients by physically motivated approximations and empirical parameters. In molecular quantum chemistry, this idea has a long history, from neglect-of-diatomic-differential-overlap (NDDO) models such as MNDO and AM1 to later, broadly applicable methods such as PM6.^1–4^ These approaches were designed primarily for isolated molecules and clusters. Their parameter sets are largely organized by elements or atomic valence states, which is one reason for their broad chemical coverage and practical appeal in molecular applications.

Empirical tight binding (ETB) or extended Hückel theory^5^ (EHT) provides another approach for SQC based on density functional theory^6^ (DFT), which has been formalized in the framework of density functional tight binding^7^ (DFTB) based on a controlled expansion around a reference density. In this construction, the density is written as ρ = ρ_0_ + δρ, where ρ_0_ is commonly built from a superposition of neutral atomic densities, and the Kohn–Sham energy is expanded in powers of the density fluctuation δρ. The zeroth- and first-order contributions lead to a non-self-consistent tight-binding Hamiltonian evaluated in the fixed reference potential, closely related to the Harris functional and to the density functional interpretation of tight-binding models.^8–11^ Inclusion of second-order and higher-order terms introduces charge fluctuation contributions for the electrostatic terms in the DFTB methods, known as self-consistent charges (SCC), which made DFTB methods more widely applicable to polar and chemically heterogeneous systems.^12,13^ While the DFTB formulation in a minimal localized orbital basis makes it an attractive choice due to its computational speed for materials, surfaces, liquids, and interfaces, the need for tabulated Slater–Koster-type integrals and pairwise repulsive parametrization kept the applicability confined to a small chemical space.^14^

Another prominent approach is extended tight binding^15,16^ (*x*TB), which replaces the numerically tabulated integrals with analytical expressions and allows for a broad element coverage by focusing on element-specific over element-pair-specific parametrization. The GFN family was constructed to deliver reliable geometries, vibrational frequencies, and noncovalent interactions over large parts of the chemical space. In its first version, the GFN1-*x*TB^17^ method builds closely on the third-order DFTB scheme but accounts effectively for many-body effects by introducing an environment dependency in many of the key parameters of the tight-binding model, like self-energies. GFN2-*x*TB further improves the physical content of the Hamiltonian through anisotropic electrostatics, including atomic dipoles and quadrupoles, together with density-dependent dispersion contributions.^18^

The GFN1-*x*TB method has mainly been applied to molecular solids^19,20^ and large-scale simulations,^21^ demonstrating the value of SQC methods for condensed-phase simulations. Periodic GFN1-*x*TB implementations are available in several electronic structure packages and employ charge-only electrostatics. Buccheri et al. recently introduced a generalized Ewald partitioning of the charge-only GFN1-*x*TB Klopman–Ohno interaction and compared it directly with smooth truncation.^22^ The DFTB+ program package exposes both GFN1-*x*TB and GFN2-*x*TB through the tblite library, while its published implementation roadmap identified the need for a full multipole Ewald summation for supporting periodic anisotropic terms of GFN2-*x*TB.^23,24^ Availability of the GFN2-*x*TB parametrization through a software interface must therefore be distinguished from a complete periodic long-range treatment of its self-consistent dipoles and quadrupoles. The present CP2K implementation supplies this multipolar treatment together with analytical forces and stress and native Bloch *k*-point sampling.

This distinction is central to periodic applications. A molecularly optimized semiempirical Hamiltonian can give excellent structures and noncovalent interaction trends for finite systems yet still show systematic errors once the same parameters are tested against cohesive energies, crystal volumes, and Brillouin-zone-converged polymorph energetics. The present CP2K implementation provides the periodic machinery to assess this transferability question quantitatively: multipolar Ewald electrostatics, *k*-point sampling, analytical stress, and crystal symmetry support.

The extension from molecular to periodic GFN2-*x*TB is nevertheless considerably more difficult than adding a conventional charge-only Ewald sum. For monopolar tight-binding models, the long-range electrostatic problem is essentially the periodic summation of atom- or shell-centered charges.^25^ GFN2-*x*TB, by contrast, uses self-consistent shell charges together with atomic dipoles and quadrupoles. The electrostatic kernel must therefore be generalized from a monopolar to a multipolar Ewald formulation, with real- and reciprocal-space terms, short-range damping, self-consistent potentials, analytical forces, and stress contributions treated consistently.^26,27^ This multipolar treatment is the central methodological step in bringing the full GFN2-*x*TB electrostatic model into periodic boundary conditions. Similarly, more recently developed tight-binding methods with anisotropic electrostatics, such as multipolar DFTB,^28^ can benefit from the present periodic multipole machinery.

Despite the remaining limitations of a molecularly parametrized model, GFN2-*x*TB has become a widely used driver for large-scale machine-learning data-set generation. It is used primarily for geometry generation and conformer searches and increasingly as a low-cost labeling level for data sets used in foundation model training.^29–31^ Multifidelity approaches that combine GFN2-*x*TB data with smaller amounts of high-fidelity reference data have also emerged as promising routes to data-efficient model training.^32^ Periodic GFN2-*x*TB in CP2K provides access to the molecular dynamics, geometry optimization, force accumulation, and stress accumulation needed for production simulations,^16,33,34^ opening the same kind of large-scale data generation for periodic systems that molecular *x*TB has enabled for finite systems. CP2K also connects GFN2-*x*TB to established simulation ecosystems such as AiiDA,^35^ i-PI,^36^ and ASE.^37^ Periodic *x*TB enables electronic structure-based simulations of molecular crystals, inorganic solids, porous materials, liquids, slabs, and interfaces at a cost that is far below conventional DFT while still retaining an electronic Hamiltonian.

In this work, we present the CP2K implementation of periodic GFN2-*x*TB based on multipolar Ewald summation. The implementation supports periodic electrostatics for the GFN2-*x*TB multipole variables, analytical forces, stress tensors for geometry and cell optimizations, explicit *k*-point sampling, and symmetry-aware crystal calculations. The method is assessed on three complementary benchmarks in the order of increasing scope: the DMC-ICE13 data set provides diffusion Monte Carlo (DMC) reference lattice energies for 13 ice polymorphs;^38^ the LC10 set of cubic covalent and ionic solids provides zero-point-corrected experimental and post-HF lattice constants and cohesive energies;^39^ and the X23b set of molecular crystals probes general intermolecular packing, relaxed-cell volumes, and cohesive energetics.^40,41^ Over the years, these data sets have provided an essential reference point for assessing newly developed periodic methods.^42–45^ Together, these tests quantify the accuracy of the current GFN2-*x*TB parametrization in periodic solids, identify where the complete GFN2-*x*TB model improves over GFN1-*x*TB, and show where the molecular parametrization is not yet sufficient for quantitatively predictive periodic cohesive energetics. GFN1-*x*TB and GFN2-*x*TB are compared throughout as their complete published parametrizations. They differ not only in the presence of anisotropic electrostatics but also in coupled Hamiltonian, repulsion, and dispersion terms; benchmark differences between them therefore cannot be attributed to a single-model component.

## 2 Theory

The purpose of this section is to separate the model ingredients that are already present in molecular GFN2-*x*TB from the terms that require genuinely periodic treatment. We first summarize the electronic structure model only to the extent needed for the present implementation and then focus on the multipolar electrostatic energy, which is the central theoretical element in the extension to periodic boundary conditions.

### 2.1 GFN2-*x*TB Model

GFN2-*x*TB is a self-consistent tight-binding method designed to retain the computational structure of a minimal-basis electronic Hamiltonian while using a broad, element-based parametrization for geometries, frequencies, and noncovalent interactions across large regions of a chemical space.^16,18^ The electronic problem is solved in a localized atomic orbital basis. The *x*TB Hamiltonian can be used in a spin-restricted formulation or, when explicit spin polarization is included, with spin-dependent coefficients.^46^ In molecular notation, the spin-dependent coefficients obey the generalized eigenvalue problem

1 $$H_{\sigma }C_{\sigma }=SC_{\sigma }\varepsilon_{\sigma }$$

where *H*_σ_ is the GFN2-*x*TB Hamiltonian, *S* is the overlap matrix, and ε_σ_ contains the orbital energies. The Hamiltonian is constructed from a short-ranged zeroth-order tight-binding part and a self-consistent response to density-dependent variables

2 $$H_{\sigma }=H^{{}^{\circ}}+\Delta H\left(\left\{q_{l}\right\},\left\{\mu_{A}\right\},\left\{\theta_{A}\right\}\right)+H^{disp}+H_{\sigma }^{spin‐pol}+H_{\sigma }^{ext}$$

Here, *q*_*l*_ are shell charges, μ_A_ are atomic dipoles, and θ_*A*_ are atomic quadrupoles derived from the current density matrix. The terms *H* ^disp^, *H*_σ_^spin‑pol^, and *H*_*σ*_^ext^ collect the density-dependent dispersion, spin polarization, and optional external potential contributions. The precise matrix construction is discussed below in the Implementation section.

The total GFN2-*x*TB energy can be written as

3 $$E_{xTB}=E_{band}+E_{rep}+E_{ES}+E_{disp}^{\left(0\right)}+E_{disp}^{SCC}+E_{spin‐pol}+E_{ext}+TS_{fermi}$$

The band energy *E*_band_ is obtained from the occupied tight-binding levels, while *E*_rep_ collects short-ranged repulsive terms, and *E*_disp_^(0)^ and *E*_disp_^SCC^ denote the zeroth-order and self-consistent dispersion contributions. The term *E*_spin‑pol_ contains the explicit spin dependence introduced in the spGFN2-*x*TB method as an on-site spin polarization contribution.^46^ The terms *TS*_Fermi_ and *E*_ext_ are included when electronic smearing or external fields are used. When fractional occupations are used, the smearing contribution follows the usual finite-temperature or broadened occupation formulations, with Fermi–Dirac occupations grounded in Mermin’s finite-temperature extension of density functional theory and Gaussian-type broadenings closely related to the standard Brillouin-zone smearing schemes used for metals and small-gap systems.^47–49^ Among these terms, *E*_ES_ is the part that requires the most substantial change when going from isolated molecules to periodic systems because GFN2-*x*TB contains monopolar, dipolar, and quadrupolar electrostatic responses. The remainder of this section, therefore, focuses on the periodic electrostatic formulation.

### 2.2 Periodic GFN2-*x*TB Electrostatics

The GFN2-*x*TB electrostatic energy is expressed in terms of shell charges *q*_*l*_, atomic dipoles *μ*_A_^*i*^, atomic quadrupoles *θ*_A_^*ij*^, and damped interaction tensors

4 $$\begin{array}{ll} E_{ES} & =\frac{1}{2}\sum_{l,l^{\prime }}q_{l}q_{l^{\prime }}T_{ll^{\prime }}+\sum_{A,B}\sum_{i}q_{A}\mu_{B}^{i}T_{AB}^{i}\\ & +\frac{1}{2}\sum_{A,B}\sum_{i,j}\mu_{A}^{i}\mu_{B}^{j}T_{AB}^{ij}+\sum_{A,B}\sum_{i,j}q_{A}\theta_{B}^{ij}T_{AB}^{ij}. \end{array}$$

Here *l*, *l*′ denote shells, A, B atoms, and *i*, *j* Cartesian components. For molecular systems, the damped shell–charge interaction is

5 $$T_{ll^{\prime }}^{mol}={\left[R_{ll^{\prime }}^{2}+\frac{1}{\eta_{ll^{\prime }}^{2}}\right]}^{-1/2}$$

where *R*_*ll*′_ is the distance between the atoms carrying shells *l* and *l*′, and η_*ll*′_ is the corresponding pair hardness.

The short-range damping used for the molecular dipole–charge, dipole–dipole, and charge–quadrupole tensors can be written compactly as

6 $$g_{AB}\left(R\right)={\left[1+6{\left(\frac{R_{A}^{c}+R_{B}^{c}}{2R}\right)}^{d}\right]}^{-1}$$

which gives

7 $$T_{AB}^{i}=g_{AB}\left(R_{AB}\right)\frac{R_{AB,i}}{R_{AB}^{3}}$$

8 $$T_{AB}^{ij}=g_{AB}\left(R_{AB}\right)\frac{\delta_{ij}R_{AB}^{2}-3R_{AB,i}R_{AB,j}}{R_{AB}^{5}}$$

Under periodic boundary conditions, the molecular kernels are replaced by Ewald sums. The construction follows the multipolar Ewald framework of Aguado and Madden and uses the compact reciprocal-space formulation and corrections of Laino and Hutter as the natural reference point for charge-, dipole-, and quadrupole-level interactions.^26,27^ For the shell–charge interaction, with $R_{ll^{\prime }}^{L}=\left|R_{l}-R_{l^{\prime }}+L\right|$ , we use

9 $$\begin{array}{ll} T_{ll^{\prime }}^{pbc} & =\sum_{L}\left[f\left(R_{ll^{\prime }}^{L}\right){\left[{\left(R_{ll^{\prime }}^{L}\right)}^{2}+\frac{1}{\eta_{ll^{\prime }}^{2}}\right]}^{-1/2}+\frac{1-f\left(R_{ll^{\prime }}^{L}\right)}{R_{ll^{\prime }}^{L}}-\frac{\mathrm{erf}\left(\alpha R_{ll^{\prime }}^{L}\right)}{R_{ll^{\prime }}^{L}}\right]\\ & +\frac{8\pi }{V}\sum_{G>0}\gamma \left(G\right)\cos\left(G\cdot R_{ll^{\prime }}\right), \end{array}$$

with

10 $$\gamma \left(G\right)=\frac{1}{G^{2}}\exp\left[-\frac{G^{2}}{4\alpha^{2}}\right]$$

where **G** is the reciprocal lattice vector. The multipolar tensors are generated consistently from the same periodic kernel, using the GFN2-specific damping of eq 6. This avoids introducing independent real- and reciprocal-space formulas for each Cartesian component and makes the self-consistent potentials straightforward, e.g.,

11 $$V_{l}^{q}=\sum_{l^{\prime }}q_{l^{\prime }}T_{ll^{\prime }}^{pbc}$$

Equivalently, the electrostatic energy can be split into direct and reciprocal parts

12 $$E_{ES}=E_{ES}^{dir}+E_{ES}^{rec}$$

The direct term contains the short-range damped real-space contributions, for example,

13 $$E_{qq}^{dir}=\frac{1}{2}\sum_{l,l^{\prime }}q_{l}q_{l^{\prime }}{\mathrm{T̃}}_{ll^{\prime }}$$

For the reciprocal-space contribution, it is convenient to introduce the structure factors

14a $$Q_{c}\left(G\right)=\sum_{A}q_{A}\;\cos\left(G\cdot R_{A}\right)$$

14b $$Q_{s}\left(G\right)=\sum_{A}q_{A}\;\sin\left(G\cdot R_{A}\right)$$

14c $$M_{c}\left(G\right)=\sum_{A}\left(G\cdot \mu_{A}\right)\cos\left(G\cdot R_{A}\right)$$

14d $$M_{s}\left(G\right)=\sum_{A}\left(G\cdot \mu_{A}\right)\sin\left(G\cdot R_{A}\right)$$

14e $$\Theta_{c}\left(G\right)=\frac{1}{3}\sum_{A}\left(G\cdot \theta_{A}G\right)\cos\left(G\cdot R_{A}\right)$$

14f $$\Theta_{s}\left(G\right)=\frac{1}{3}\sum_{A}\left(G\cdot \theta_{A}G\right)\sin\left(G\cdot R_{A}\right)$$

The reciprocal electrostatic energy is then

15a $$E_{qq}^{rec}=\frac{4\pi }{V}\sum_{G>0}^{G_{\max}}\gamma \left(G\right)\left[Q_{c}{\left(G\right)}^{2}+Q_{s}{\left(G\right)}^{2}\right]$$

15b $$E_{q\mu }^{rec}=\frac{8\pi }{V}\sum_{G>0}^{G_{\max}}\gamma \left(G\right)\left[Q_{s}\left(G\right)M_{c}\left(G\right)-Q_{c}\left(G\right)M_{s}\left(G\right)\right]$$

15c $$E_{\mu \mu }^{rec}=\frac{4\pi }{V}\sum_{G>0}^{G_{\max}}\gamma \left(G\right)\left[M_{c}{\left(G\right)}^{2}+M_{s}{\left(G\right)}^{2}\right]$$

15d $$E_{q\theta }^{rec}=\frac{8\pi }{V}\sum_{G>0}^{G_{\max}}\gamma \left(G\right)\left[Q_{c}\left(G\right)\Theta_{c}\left(G\right)+Q_{s}\left(G\right)\Theta_{s}\left(G\right)\right]$$

## 3 Implementation

The implementation is designed as a CP2K integration using the tblite library for providing the GFN2-*x*TB model ingredients, while the periodic simulation machinery, electronic minimization, force accumulation, and large-scale parallel execution remain under the CP2K control. The following subsections, therefore, describe how the two codes exchange the image-resolved Hamiltonian, overlap, density, energy, force, and stress information needed for geometry optimizations and molecular dynamics under periodic boundary conditions.

### 3.1 CP2K/tblite Interface

The periodic GFN2-*x*TB implementation couples CP2K’s electronic structure infrastructure to the tblite library. The model-specific ingredients, such as element parameters, repulsive potentials, coordination number-dependent terms, anisotropic electrostatics, and the self-consistent D4 contribution, are evaluated by tblite, whereas CP2K provides the periodic simulation cell, sparse matrix storage, density matrix optimization, molecular dynamics drivers, and the accumulation of forces and virials. This separation keeps the GFN2-*x*TB Hamiltonian close to its reference formulation^18^ while making it available in the same simulation environment as the other CP2K force methods.^16,33,34^

A practical advantage of this embedding is that periodic GFN2-*x*TB can use CP2K’s existing electronic minimization and solver infrastructure. For moderate system sizes, this includes the orbital transformation minimizer^50^ as well as conventional diagonalization with distributed eigensolvers such as ELPA, DLA-Future, and cuSOLVERMp, including GPU-enabled variants where available.^51–53^ For very large systems, the same interface can be coupled to CP2K’s linear-scaling density matrix machinery^54–56^ and, in particular, to the non-orthogonal local submatrix method used in our recent large-scale GFN1-*x*TB work.^57,58^ This submatrix route has enabled periodic GFN1-xTB simulations with up to 102 million atoms and subsequent GPU-based exascale demonstrations. Although the present benchmarks are much smaller, retaining these solver paths is important because the target applications of periodic GFN2-*x*TB are long trajectories and large condensed-phase models rather than isolated single-point calculations.^59–61^

### 3.2 Real-Space Hamiltonian Construction

CP2K initializes the tblite molecular container from the nuclear positions, the lattice vectors, and the periodicity flags of the active simulation cell. For a localized atomic orbital χ_μA_ on atom *A* and the image of χ_νB_ translated by the lattice vector **L**, the overlap matrix is stored in real space as

16 $$S_{\mu A,\nu B}^{L}=\left\langle \chi_{\mu A}|\chi_{\nu B}^{L}\right\rangle$$

The corresponding zeroth-order GFN2-*x*TB Hamiltonian block has the usual extended Hückel form

17 $$H_{\mu A,\nu B}^{0,L}=\frac{1}{2}\left(\varepsilon_{l_{\mu }A}+\varepsilon_{l_{\nu }B}\right)k_{l_{\mu }l_{\nu }}^{AB}f_{l_{\mu }l_{\nu }}^{AB}\left(R_{AB}^{L}\right)S_{\mu A,\nu B}^{L}$$

where $\varepsilon_{lA}$ denotes the shell energy, $k_{l_{\mu }l_{\nu }}^{AB}$ is the element- and angular-momentum-dependent Wolfsberg-Helmholtz factor, and $f_{l_{\mu }l_{\nu }}^{AB}$ collects the distance-dependent scaling functions. All pairs within the tblite neighbor list are assembled as image blocks, so that the same real-space representation can be used for isolated, partially periodic, and fully periodic calculations.

Self-consistency enters through a potential matrix that is rebuilt from the current shell populations and atomic multipoles. In compact notation, CP2K adds to each image block

18 $$\begin{array}{ll} \Delta H_{\mu A,\nu B,\sigma }^{L} & =-\frac{1}{2}\left[\left(v_{A\sigma }^{at}+v_{B\sigma }^{at}\right)S_{\mu A,\nu B}^{L}+{\left(v_{A\sigma }^{sh}S^{L}+S^{L}v_{B\sigma }^{sh}\right)}_{\mu \nu }\right]\\ & -\frac{1}{2}\sum_{i}\left[v_{Ai,\sigma }^{\mu }D_{\mu A,\nu B;i}^{A,L}+v_{Bi,\sigma }^{\mu }D_{\mu A,\nu B;i}^{B,L}\right]\\ & -\frac{1}{2}\sum_{ij}\left[v_{Aij,\sigma }^{\theta }Q_{\mu A,\nu B;ij}^{A,L}+v_{Bij,\sigma }^{\theta }Q_{\mu A,\nu B;ij}^{B,L}\right]. \end{array}$$

Here, *v* ^at^ and **v** ^sh^ are the atom- and shell-resolved charge potentials, while v^μ^ and v^θ^ are the dipolar and quadrupolar potentials generated by the GFN2-*x*TB anisotropic electrostatic response. The matrices *D* and *Q* are the corresponding dipole and quadrupole operator integrals in the local *x*TB basis. The spin index σ is retained because the current interface supports both restricted and unrestricted calculations; for unrestricted calculations, CP2K propagates charge and magnetization variables consistently through the same machinery.

After diagonalization or direct density matrix minimization, the energy decomposition in eq 3 is reported to CP2K. This is the quantity differentiated by the force infrastructure and used by molecular dynamics, geometry optimization, and free-energy calculations.

### 3.3 Periodic Boundary Conditions

For periodic systems CP2K stores the overlap and Hamiltonian in an image-resolved real-space representation and passes the periodicity mask to tblite. The same machinery covers fully periodic molecular crystals as well as partially periodic slabs, interfaces, and one-dimensional models. For calculations beyond the Γ-point, the image matrices are transformed to the Bloch form

19 $$H_{\sigma }\left(k\right)=\sum_{L}e^{ik\cdot L}\left(H^{0,L}+\Delta H_{\sigma }^{L}\right),,S\left(k\right)=\sum_{L}e^{ik\cdot L}S^{L}$$

The generalized eigenvalue problem is solved independently at each sampled crystal momentum, and the image-resolved density matrix is reconstructed as

20 $$P_{\sigma }^{L}=\sum_{k}w_{k}e^{-ik\cdot L}C_{\sigma }\left(k\right)f_{\sigma }\left(k\right)C_{\sigma }^{†}\left(k\right),\;\sum_{k}w_{k}=1$$

The reconstructed *P*_σ_^**L**^ is then contracted with the same overlap, dipole, and quadrupole operator blocks that enter the self-consistent GFN2-*x*TB potential. Thus, Mulliken shell charges, atomic dipoles, and atomic quadrupoles are evaluated with the same *k*-point sampling as the band energy. The CP2K implementation supports Monkhorst–Pack grids, MacDonald shifted meshes, and explicitly supplied closed *k*-point sets for the diagonalization route.^62,63^ Symmetry-equivalent *k* points can be reduced with CP2K’s established *k*-point machinery or with the spglib-based crystal symmetry backend;^64^ the calculations reported here use full symmetry reduction, including the fractional translations of nonsymmorphic operations. The ice polymorph (DMC-ICE13) calculations reported below use native Bloch meshes with full symmetry reduction throughout. Full symmetry reduction is part of the implementation and is particularly important for larger cells, denser meshes, and less favorable parallel decompositions. Additional implementation details are given in the Supporting Information.

### 3.4 Self-Consistent Variables and Mixing

Tight-binding models provide an advantage when obtaining the self-consistent solution to the Hamiltonian, since the number of self-consistent variables is limited to the density populations rather than the full-density matrix as in other electronic structure methods, like DFT. In monopolar tight-binding methods, this is limited to the atomic or shell populations, while for GFN2-*x*TB, additionally, the atomic dipole and quadrupole populations need to be considered. In many realistic cases, the number of shell charges, atomic dipoles, and atomic quadrupoles is smaller than the whole density matrix and, therefore, more memory-efficient than keeping a history of larger density matrices. CP2K can either mix this full-variable vector through its tight-binding infrastructure or delegate the fixed-point update to the native tblite mixer. These history-dependent accelerators are closely related to the quasi-Newton ideas of Broyden and to Pulay’s direct inversion in the iterative subspace (DIIS), with modified Broyden variants being widely used for electronic structure self-consistency iterations.^65–68^ For density matrix optimization routes, including orbital transformation,^50^ the separate SCC-variable mixer is bypassed, and the GFN2-*x*TB variables are updated directly from the current density. The explicit variable definitions and update equations are given in the Supporting Information.

### 3.5 Forces, Virial, and Stress

Analytical forces are obtained by differentiating both the explicit tblite energy terms and the image-resolved Hamiltonian and overlap matrices. At self-consistency, the force on atom *I* can be written schematically as

21 $$F_{I}=-\frac{\partial E_{rep+disp}}{\partial R_{I}}-\sum_{\sigma ,L}Tr\left[P_{\sigma }^{L}\frac{\partial H_{\sigma }^{L}}{\partial R_{I}}-W_{\sigma }^{L}\frac{\partial S^{L}}{\partial R_{I}}\right]$$

where *W*_σ_^**L**^ is the energy-weighted density matrix. The first term contains the derivatives returned by tblite, including repulsion, dispersion, and halogen-bond interaction contributions where applicable. The second term contains the Pulay and Hamiltonian response contributions assembled by CP2K, including derivatives of the shell charges and multipole response operators. This form has the usual Hellmann–Feynman part associated with the explicit Hamiltonian derivative and the Pulay correction associated with the derivative of the nonorthogonal atomic orbital basis and overlap matrix.^69–71^ Because the self-consistent shell charges, dipoles, and quadrupoles are stationary at convergence, additional non-self-consistent response terms vanish at self-consistency, analogous to the simultaneous density geometry relaxation arguments of Bendt and Zunger and the second-generation Car-Parrinello formulation of Kühne et al.^59,72^

The stress tensor follows from the derivative with respect to a homogeneous cell deformation. This is the same variational stress setting as in the first-principles stress formalism of Nielsen and Martin, which relates energy derivatives, virials, and forces in periodic electronic structure calculations.^73,74^ CP2K accumulates the tblite tensor **σ** ^*x*TB^ into its virial convention as

22 $$W_{CP2K}^{xTB}=-\sigma^{xTB},\;\Pi^{xTB}=\frac{1}{V}W_{CP2K}^{xTB}$$

For periodic applications, the stress contribution is required for reliable CELL_OPT calculations, variable-cell molecular dynamics,^75,76^ and equation-of-state benchmarks. When a cell optimization should preserve the initial crystal symmetry or space group, CP2K can use its KEEP_SPACE_GROUP procedure: the space group is detected with the spglib library, and the resulting symmetry operations are used to keep the optimized structure in the requested symmetry setting.^64^ This is particularly useful for symmetry-constrained CELL_OPT calculations and for calculations in which the stress is accumulated from molecular dynamics sampling because small numerical distortions should not drive the cell into an unintended lower-symmetry representation. Together with the analytical forces in eq 21, it also makes geometry optimization and stress-converged structure searches possible with the same GFN2-xTB Hamiltonian used for the final energy evaluation.

## 4 Results and Discussion

This section evaluates periodic GFN-xTB across three complementary regimes. DMC-ICE13 probes k-point-converged relative energies of hydrogen-bonded ice polymorphs, LC10 tests equilibrium lattice constants and cohesive energies of compact covalent and ionic solids, and X23b assesses relaxed-cell volumes and lattice energies across chemically diverse molecular crystals. Together, these benchmarks distinguish numerical convergence from model error and structural transferability from cohesive-energy accuracy.

### 4.1 Benchmark Strategy

The individual benchmark protocols and reference quantities are defined below. The DMC-ICE13 benchmark provides a focused and stringent first test for hydrogen-bonded molecular crystals.^38^ Because the data set contains ambient and high-pressure ice polymorphs with diffusion Monte Carlo reference lattice energies, it probes whether the periodic GFN2-xTB electrostatics and dispersion terms produce a physically meaningful relative stability across structurally distinct hydrogen-bond networks. This is particularly relevant for the present implementation, since the ice polymorphs are sensitive to the long-range electrostatic treatment that motivated the multipolar Ewald formulation. In the present single-point benchmark, we focus on relative polymorph energies with respect to ice Ih because this quantity is obtained directly from periodic bulk calculations and does not require an additional isolated water monomer reference. The resulting DMC-ICE13 relative-energy mean absolute error (MAE) is 3.46 kJ mol^–1^ for GFN2-*x*TB and 8.01 kJ mol^–1^ for GFN1-*x*TB on the Γ-centered 3^3^ *k*-point mesh when evaluated over the 12 nonreference polymorphs.

The LC10 benchmark then extends the assessment to ten small cubic covalent and ionic solids selected from ref 39. We determine lattice constants from native Bloch 4^3^-mesh equations of state with full symmetry reduction in conventional eight-atom cells and evaluate cohesive energies with matching isolated atom references. Native Bloch 3^3^, 4^3^, and 5^3^ single points with full symmetry reduction establish the *k*-point convergence of the reported cohesive energies. LC10 deliberately provides a compact main-group test and does not establish robustness for transition-metal oxides, mixed-valence compounds, or multicomponent materials with more difficult charge-transfer behavior. Such systems remain outside the present benchmark scope and are an important limitation for future assessment of molecularly parametrized *x*TB models.

Finally, the X23b molecular crystal benchmark combines cohesive energetics with fully relaxed unit cell volumes for small organic molecular crystals spanning different dominant intermolecular interactions.^40,41^ The recently reported diffusion Monte Carlo lattice energies for all X23 molecular crystals provide an independent high-accuracy electronic structure scale and highlight that experimentally inferred lattice energies can carry non-negligible reference uncertainties.^77^ We therefore use the X23b values as the primary reference for the present zero-temperature volume and lattice-energy assessment and discuss the DMC-X23 results as additional context for the expected reference scale. The resulting X23b statistics are reported in Table 2.

**Table 1:** LC10 Equation-of-State Errors Relative to Zero-Point-Corrected Experiment^a^

method	*N*	ME(*a*)	MAE(*a*)	ME(*E*_coh_)	MAE(*E*_coh_)
HF	10	0.052	0.056	–1.623	1.623
MP2	10	–0.009	0.018	0.248	0.248
SCS-MP2	10	0.006	0.011	0.031	0.063
SOS-MP2	10	0.014	0.014	–0.073	0.089
GFN1-*x*TB	10	0.022	0.145	1.467	1.544
GFN2-*x*TB	10	–0.006	0.062	1.107	1.299

a lattice-constant errors are in Å and cohesive-energy errors are in eV per atom. *N* is the number of solids.

**Table 2:** Aggregate X23b Errors for Native Bloch 2^3^-Mesh Crystal Cell Optimizations with Full Symmetry Reduction and 3^3^ Lattice-Energy Re-Evaluation on the Optimized Cells^a^

quantity	method	*N*	ME	MAE	RMSE	MaxAE
lattice energy	GFN1-*x*TB	23	0.26	11.35	14.02	30.94
lattice energy	GFN2-*x*TB	23	–12.02	14.09	21.34	77.79
lattice energy	DMC-X23	23	–1.99	3.72	4.69	10.80
lattice energy	ML-LNO–CCSD(T)/RPA + ph	23	0.82	2.63	3.34	8.90
cell volume	GFN1-*x*TB	23	–5.96	7.51	9.02	19.24
cell volume	GFN2-*x*TB	23	–1.66	5.84	7.53	19.95
cell volume	PBE + D3	23	4.50	4.50	5.14	13.11
cell volume	BLYP + D3	23	0.89	1.97	2.43	6.67
cell volume	RPBE + D3	23	6.63	6.63	7.77	19.72

a Lattice-energy errors are reported in kJ mol^–1^ per molecule and volume errors in percent relative to the X23b electronic reference volumes. *N* gives the number of converged relaxed-cell entries included in each statistic. The DMC-X23 and ML-LNO–CCSD­(T)/RPA + ph rows are independent high-level electronic lattice energies from refs 77,78 , evaluated here against X23b. The DFT + D3 volume rows are computed from the electronic volumes reported in ref 41

We separate structural correctness from cohesive-energy accuracy, since an inexpensive periodic method that yields reasonable cell volumes and robust pre-optimized structures represents a valuable tool for screening and large-scale exploratory simulations. The combined structural and energetic metrics, therefore, establish a practical accuracy range for the current implementation and highlight its effectiveness for periodic modeling. The numerical validation of the new multipolar implementation is kept separate from this accuracy assessment and is based on finite-difference forces and stresses and on CP2K–CLI agreement, as documented in the Supporting Information.

### 4.2 DMC-ICE13 Ice Polymorphs

The DMC-ICE13 benchmark provides a demanding first test of periodic GFN2-*x*TB for hydrogen-bonded molecular crystals. The most diagnostic quantity is not simply the absolute lattice energy of each phase but whether the method preserves the small relative-energy differences that determine the ice polymorph ordering. We therefore performed CP2K/tblite single-point calculations for all 13 DMC-ICE13 reference geometries using periodic GFN1-*x*TB and GFN2-*x*TB. To match the Brillouin-zone sampling used for the published DMC-ICE13 DFT benchmark as closely as possible, we evaluated Γ-point-only calculations and native Bloch meshes with full symmetry reduction using Γ-centered 2^3^, 3^3^, 4^3^, and 5^3^ *k*-point meshes. The main comparison below uses the 3^3^ mesh, which is the mesh used for the published GGA, *meta*-GGA, and vdW-inclusive single-point DFT calculations; the corresponding *k*-point dependence is summarized in the Supporting Information. The reported energy differences are per water molecule and referenced to ice Ih

23 $$\Delta E_{\alpha }=\frac{E_{\alpha }}{N_{\alpha }}-\frac{E_{Ih}}{N_{Ih}}$$

where *N*_α_ is the number of water molecules in polymorph α. The MAE, mean error (ME), root-mean-square error (RMSE), and maximum absolute error (MaxAE) quoted below are evaluated over the 12 nonreference phases, so that the zero error of ice Ih is not included by construction.

The *k*-point dependence shows why Γ-point-only calculations are not an appropriate basis for comparison with the published DFT data. The Γ-point-only numbers give MAEs of 6.69 and 5.58 kJ mol^–1^ for GFN1-*x*TB and GFN2-*x*TB, respectively, but they differ noticeably from the converged Brillouin-zone-sampled results for several dense phases, most pronounced in the case of ice VII. For ice VII, the GFN2-*x*TB relative-energy error decreases from +33.46 kJ mol^–1^ at the Γ point to +3.41 kJ mol^–1^ on the converged 3^3^ mesh. This unusually large Γ-point sensitivity is likely a consequence of its dense, highly periodic high-pressure structure. The short intermolecular separations increase the dispersion of the *x*TB band energy and make the self-consistent charge and multipole response more sensitive to Brillouin-zone sampling. As a result, Γ-point sampling can represent a particularly poor approximation for this phase, whereas explicit *k*-point integration removes the corresponding phase-specific aliasing. Any apparently better Γ-point-only agreement is therefore counterintuitive only if the benchmark agreement is confused with *k*-point convergence. In the present case, the Γ-point-only result can benefit from fortuitous cancellation between the Brillouin-zone sampling error and the intrinsic GFN-*x*TB model error; using it would make the convergence behavior of GFN-*x*TB appear more benign than it is for periodic ice energetics. The aggregate Γ-point diagnostics are reported in Figure S1 and Table S1 of the Supporting Information. The 2^3^ mesh is already close to the converged aggregate statistics, changing the MAE relative to 3^3^ by only about 0.05 kJ mol^–1^, but individual relative energies still shift by up to 0.58 kJ mol^–1^. The *k*-point mesh is effectively converged at 3^3^, since larger meshes such as 4^3^ and 5^3^ change the scale of errors on DMC-ICE13 by less than 0.01 kJ mol^–1^, and the 3^3^-to-5^3^ MAE change is below 0.002 kJ mol^–1^. The comparison thus underscores the importance of explicit *k*-point sampling, and for larger applications of the symmetry reduction machinery described above, even for a low-cost Hamiltonian such as GFN-*x*TB.

The phase-resolved errors in Figure 1 show that GFN2-*x*TB is a clear improvement over GFN1-*x*TB for this benchmark, but that the improvement is not sufficient to reproduce the DMC ordering. The corresponding numerical values are tabulated in Table S2 of the Supporting Information. At the 3^3^ *k*-point level, GFN1-*x*TB systematically overstabilizes the non-Ih polymorphs, with a mean signed error of −8.01 kJ mol^–1^ and the largest error for ice VIII (−13.66 kJ mol^–1^). GFN2-*x*TB reduces the relative-energy MAE from 8.01 to 3.46 kJ mol^–1^ and the RMSE from 8.65 to 3.81 kJ mol^–1^. This improvement is chemically meaningful, but several dense phases remain too stable relative to ice Ih, and the residual signed error is still −2.84 kJ mol^–1^. Because all DMC-ICE13 values considered here are referenced to ice Ih, these signed relative-energy errors do not establish a general underbinding or overbinding bias in the absolute lattice energies; instead, they show that the non-Ih phases are on average stabilized too strongly relative to Ih. The largest GFN2-*x*TB deviation is ice XIII (−6.35 kJ mol^–1^), followed by ice VI and XV. Thus, the residual error is not a simple constant shift that could be removed by referencing; it reflects a phase-dependent imbalance between the hydrogen-bond network, short-range repulsion, dispersion, and multipolar electrostatics. For dense high-pressure phases, especially ice VII and VIII, the relevant balance involves hydrogen-bond directionality, short-range O–O repulsion, density-dependent dispersion, and the self-consistent multipolar response. These phases should therefore be included explicitly in any periodic or condensed-phase refit, rather than relying only on molecular water clusters or ambient pressure liquid structures. The goal would not be to introduce a phase-specific correction but to remove the systematic overstabilization of compact hydrogen-bond networks while preserving the transferability that makes *x*TB useful.

**Figure 1:**
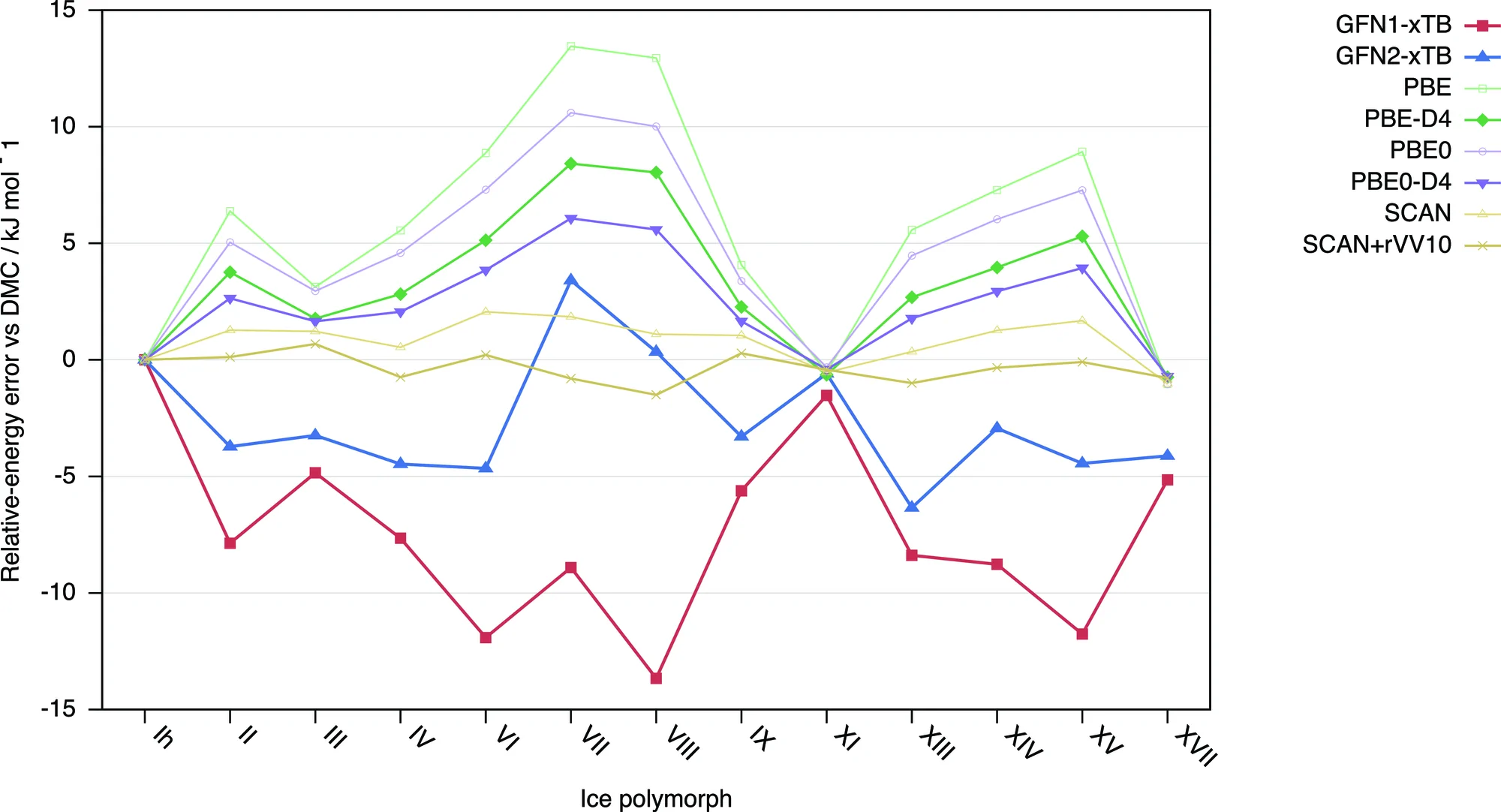
Phase-resolved DMC-ICE13 relative energy errors for periodic GFN1-*x*TB and GFN2-*x*TB compared with the representative density functional approximations. The plotted quantity is Δ*E*_method_ – Δ*E*_DMC_ for each ice polymorph, with ice Ih as the common zero of relative energy, the horizontal line marks exact agreement with the DMC reference, whereas negative values indicate polymorphs that are predicted too stable relative to ice Ih. Solid GFN curves denote CP2K/tblite single-point calculations on the DMC-ICE13 reference geometries using a Γ-centered 3^3^ *k*-point mesh. The DFT data are the published DMC-ICE13 values of ref 38; PBE/PBE-D4, PBE0/PBE0-D4, and SCAN/SCAN + rVV10 are shown as paired uncorrected and dispersion- or nonlocal-correlation-corrected references.

The comparison with the existing DMC-ICE13 DFT data in Figure 1 provides a better perspective for this result. The best published density functional approximations for relative energies, such as optB86b-vdW, B3LYP-D3atm, SCAN + rVV10, and revPBE-D3, achieve sub-kJ mol^–1^ to about 0.8 kJ mol^–1^ MAEs on the same relative-energy metric. Many standard dispersion-corrected and nonlocal functionals fall in the range of 1–4 kJ mol^–1^. Periodic GFN2-*x*TB does not yet reach the accuracy of the best DFT descriptions of ice polymorph energetics, and on this fixed-geometry relative-energy test, several DFT references are closer to the DMC values. The benchmark of the ice polymorphs shows a clear improvement of the complete GFN2-*x*TB model over GFN1-*x*TB on this relative-energy metric. Because the two methods differ in several coupled terms, the improvement should not be assigned specifically to multipolar electrostatics. However, the energetic ordering of the ice polymorphs is not reproduced quantitatively with GFN-*x*TB methods, highlighting potential issues for using molecular parametrizations for condensed matter.

### 4.3 LC10 Covalent and Ionic Solids

Following the molecular ice test, LC10 probes a qualitatively different regime: extended covalent networks and strongly ionic crystals with compact primitive cells. Table 1 compares the native Bloch CP2K/tblite equations of state with the zero-point-corrected experimental and post-HF values of Goldzak et al.^39^ All ten systems yield valid minima with both GFN methods. The complete GFN2-*x*TB model reduces the lattice-constant MAE from 0.145 to 0.062 Å and the cohesive-energy MAE from 1.544 to 1.299 eV per atom relative to GFN1-*x*TB, although the cohesive energy errors remain much larger than the SCS-MP2 and SOS-MP2 references. The corresponding system-resolved lattice constants and cohesive energies are reported in Table S3 of the Supporting Information.

### 4.4 X23b Molecular Crystals

The final and broadest benchmark, X23b, establishes how transferable periodic GFN2-*x*TB is across organic molecular crystals. The optimized structures should first be compared against the electronic reference cell volumes because a reliable periodic method must place molecules at reasonable separations before the lattice energies can be interpreted. Across the full X23b set, native Bloch 2^3^-mesh cell optimizations with full symmetry reduction and an angle-conserving cell optimization protocol give a mean absolute volume error of 5.84% for GFN2-*x*TB, compared with 7.51% for GFN1-*x*TB. The mean signed volume error is −1.66% for GFN2-*x*TB, but the residual errors are system-dependent, with the largest GFN2-*x*TB volume deviations for adamantane, anthracene, imidazole, and naphthalene. Thus, the periodic GFN2-*x*TB implementation yields physically reasonable relaxed molecular crystal cells for many X23b systems, but the structural accuracy is not uniform across packing motifs. For direct context, the fully relaxed electronic DFT + D3 volumes reported by ref 41 for the same X23b construction give mean absolute volume errors of 4.50%, 1.97%, and 6.63% for PBE + D3, BLYP + D3, and RPBE + D3, respectively.

The lattice-energy comparison is more stringent. Using the same 2^3^-mesh cell-optimized structures and re-evaluating the lattice energies on the 3^3^ mesh, the X23b lattice-energy MAE is 14.09 kJ mol^–1^ for GFN2-*x*TB and 11.35 kJ mol^–1^ for GFN1-*x*TB; the corresponding RMSEs are 21.34 and 14.02 kJ mol^–1^, respectively. Explicit k-point-sampled relaxation, therefore, substantially reduces the absolute X23b lattice-energy errors relative to Γ-point relaxation, but the aggregate improvement from GFN1-*x*TB to GFN2-*x*TB is not systematic for cohesive energetics, even though the volume statistics improve more clearly. The dominant GFN2-*x*TB lattice-energy outliers are imidazole, naphthalene, adamantane, hexamine, and pyrazole. In particular, the strong overbinding of the polycyclic aromatic crystals indicates that the present transferable molecular parametrization does not provide a balanced description of dispersion-dominated molecular crystal cohesion when applied without periodic refitting. The Supporting Information reports both the fixed-reference-geometry diagnostic and the final-relaxed-geometry *k*-point convergence test. On the final cells, the mean absolute energy change from the 3^3^ to the 4^3^ mesh is only 0.079 and 0.084 kJ mol^–1^ for GFN1-*x*TB and GFN2-*x*TB, respectively. The reported cells, therefore, come from fully k-point-sampled native Bloch optimization rather than post-processing Γ-optimized geometries, while the 3^3^ energy re-evaluation removes the remaining integration error of the 2^3^ optimization mesh.

This pattern suggests concrete targets for improvement. A periodic reparametrization should explicitly constrain the dispersion–repulsion balance for π-stacked and other aromatic packing motifs while also retaining polar and hydrogen-bonded molecular crystals in the training set. Fitting lattice energies alone would be risky: the X23b volume errors show that cohesive-energy improvements should be optimized together with relaxed-cell volumes, and ideally with periodic forces and stresses, to avoid compensating errors between packing geometry and interaction strength.

The system-resolved profile in Figure 2 makes this point more directly than the aggregate statistics: the DMC-X23 and ML-LNO–CCSD­(T)/RPA + ph values remain close to the X23b reference scale for most systems, whereas the GFN-*x*TB deviations are strongly system-dependent and dominated by a small number of molecular packing motifs.

**Figure 2:**
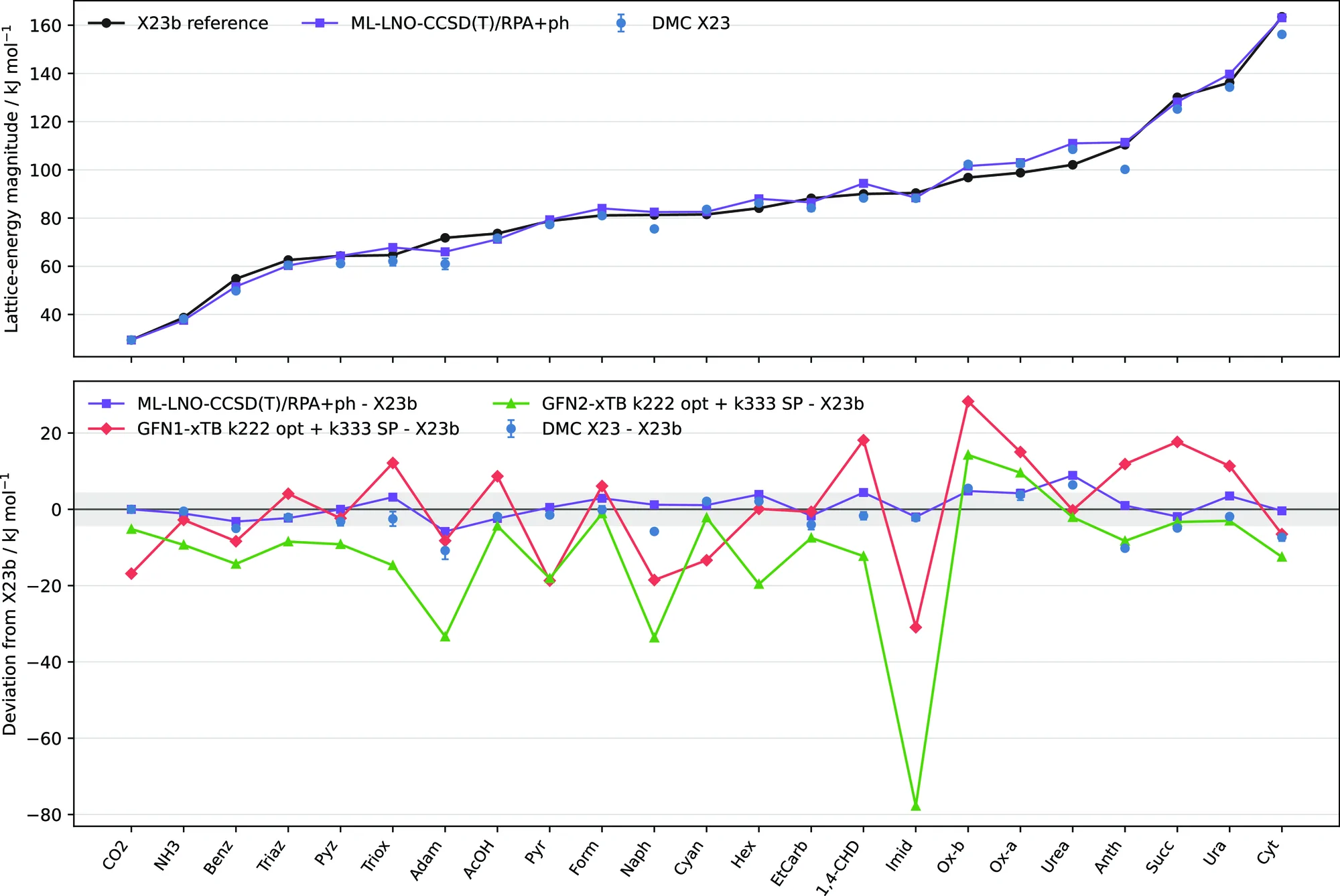
System-resolved X23b lattice-energy benchmark for periodic GFN1-*x*TB and GFN2-*x*TB. The upper panel shows the X23b reference lattice-energy magnitudes together with diffusion Monte Carlo (DMC) X23 values from ref. 77 and ML-LNO–CCSD­(T)/RPA + ph lattice energies from ref 78, which provide independent high-accuracy reference scales for the same molecular crystal family. Systems are ordered by increasing X23b reference lattice energy. The lower panel reports deviations from the X23b reference for native Bloch 3^3^ single-point energies on the 2^3^ mesh cell-optimized periodic CP2K/tblite structures and for the high-level electronic references. The gray band marks ±4.184 kJ mol^–1^; the DMC–X23b and ML-LNO–CCSD­(T)/RPA + ph–X23b differences contextualize the reference scale rather than serving as additional targets used in the present optimization.

Consequently, periodic GFN2-*x*TB is well-suited for inexpensive molecular crystal pre-optimization, structural screening, and generating physically reasonable starting points for higher-level calculations.

## 5 Conclusions

We have presented a periodic GFN2-*x*TB implementation in CP2K based on a consistent multipolar Ewald treatment of the charge, dipole, and quadrupole degrees of freedom in the GFN2-*x*TB electrostatic model. The implementation preserves the atom-centered parametrization strategy that makes *x*TB attractive for chemically diverse systems while adding the periodic infrastructure needed for molecular crystals, ice polymorphs, liquids, interfaces, and extended materials. Analytical forces, stress tensors, *k*-point sampling, and symmetry-aware crystal calculations make the method applicable not only to single-point energies but also to geometry optimization, cell optimization, equation-of-state calculations, and variable cell molecular dynamics.

The benchmarks provide a deliberately balanced picture. For DMC-ICE13 single-point calculations on the reference geometries, GFN2-*x*TB improves relative ice polymorph energetics over GFN1-*x*TB, reducing the MAE from 8.01 to 3.46 kJ mol^–1^ on the Γ-centered 3^3^ *k*-point mesh. Within DMC-ICE13, the remaining errors are strongly phase-dependent, with ice XIII, VI, and XV among the prominent outliers, and the DMC ordering of the low-energy polymorphs is not fully recovered. Explicit Brillouin-zone sampling is essential for a meaningful assessment: Γ-point-only DMC-ICE13 calculations can change the apparent accuracy through error compensation, whereas 3^3^, 4^3^, and 5^3^ meshes are converged on the scale of the benchmark errors. For LC10, GFN2-*x*TB improves both the lattice-constant and cohesive-energy errors relative to GFN1-*x*TB across the common ten-solid set. For X23b, native Bloch 2^3^-mesh cell optimizations give physically reasonable but nonuniform relaxed structures, with a GFN2-*x*TB cell volume MAE of 5.84%; however, the lattice-energy MAE after 3^3^ re-evaluation on these cells remains sizeable, at 14.09 kJ mol^–1^ for the full relaxed-cell set.

Taken together, the results define the current strengths of periodic GFN2-*x*TB as a low-cost electronic structure approach for structural screening, pre-optimization, and large exploratory simulations of molecular solids. For final quantitative periodic cohesive energetics, GFN2-*x*TB is best viewed as complementary to higher-level references rather than as a replacement for them. With periodic GFN2-*x*TB, *k*-point sampling, and symmetry-reduced crystal procedures available in CP2K, future GFN-*x*TB parameters can be trained directly against molecular crystals, ice polymorphs, and condensed-phase reference data rather than being transferred unchanged from finite molecular systems.

The most direct route forward is therefore a periodic training set that combines molecular crystal lattice energies, relaxed-cell metrics, ice polymorph relative energies, and periodic forces and stresses. Such a fit would test whether the present deviations can be reduced by rebalancing repulsion, dispersion, and multipolar electrostatics in extended systems, rather than by post-processing or by accidental cancellation of *k*-point and model errors.

## Supplementary Material



## Data Availability

The data supporting the findings of this study are available within the article and its Supporting Information. The DMC-ICE13, LC10, and X23b CP2K input files, CP2K outputs, analysis scripts, tabulated benchmark data, and figure-generation scripts are curated at https://github.com/DCM-Uni-Paderborn/Periodic-GFN2-Benchmarks

## Notes

Additional implementation and numerical-validation details; DMC-ICE13, LC10, and X23b benchmark data and *k*-point convergence tests (PDF)
