# Supplementary material for: Periodic GFN2‑xTB in CP2K: Multipolar Ewald Electrostatics, k‑Point Sampling, and Transferability Benchmarks for Solids
Source: J Chem Theory Comput. 2026 Sep 8;22(18):9688–99. doi: 10.1021/acs.jctc.6c01034 (PMC13602927; doi:10.1021/acs.jctc.6c01034)
Supplement: Supplementary file 1 [file ct6c01034_si_001.pdf]

Supporting Information: Periodic GFN2-xTB in CP2K: Multipolar Ewald Electrostatics, k-Point Sampling, and Transferability Benchmarks for Solids

Vahideh Alizadeh,[†,‡,⊥] Johann Pototschnig,[†,‡,⊥] Leopold M. Seidler,[¶] Sebastian Ehlert,[§] and Thomas D. Kühne[*,†,‡,‖]

†*Center for Advanced Systems Understanding (CASUS), Conrad-Schiedt-Straße 20, 02826 Görlitz, Germany*

‡*Helmholtz Zentrum Dresden-Rossendorf, Bautzner Landstraße 400, 01328 Dresden, Germany*

¶*Mulliken Center for Theoretical Chemistry, Universität Bonn, Beringstraße 4, 53115 Bonn, Germany*

§*AI for Science, Microsoft Research, Karl-Liebknecht-Str. 32, 10178 Berlin, Germany*

‖*Institute of Artificial Intelligence, Technische Universität Dresden, Helmholtzstraße 10, 01069 Dresden, Germany*

⊥*These authors contributed equally to this work.*

E-mail: tkuehne@cp2k.org

1 Self-Consistent Variables and Mixing

The self-consistent variable space of periodic GFN2-xTB is larger than in charge-only tight-binding models. For each atom, the CP2K interface collects the variables

$$\mathbf{x}_A = \left(q^{\mathrm{sh}}_{A1}, \ldots, q^{\mathrm{sh}}_{Am_A}, \mu_{Ax}, \mu_{Ay}, \mu_{Az}, \theta_{A1}, \ldots, \theta_{A6}\right), \tag{1}$$

where the first block contains the shell charges, followed by the Cartesian dipole and the six independent Cartesian quadrupole components. In spin polarized calculations the alpha and beta variables are recast into charge and magnetization channels,

$$\mathbf{x}^{\mathrm{ch}}_A = \mathbf{x}^{\alpha}_A + \mathbf{x}^{\beta}_A, \qquad \mathbf{x}^{\mathrm{mag}}_A = \mathbf{x}^{\alpha}_A - \mathbf{x}^{\beta}_A. \tag{2}$$

The CP2K-side mixer therefore no longer acts only on charges, but on the full vector of shell charges, dipoles, quadrupoles, and, when present, their magnetization counterparts. Alternatively, the native modified-Broyden TBLITE mixer can be used during conventional diagonalization. The underlying fixed-point acceleration follows the same logic as Broyden's quasi-Newton update and Pulay's DIIS extrapolation, with Johnson's modified Broyden scheme providing the commonly used electronic-structure variant of the method.[1–4] Both routes are fixed-point iterations of the form

$$\mathbf{x}^{(n+1)} = \mathcal{M}\left[\mathbf{x}^{(n)}, \mathbf{x}[P^{(n)}] - \mathbf{x}^{(n)}\right], \tag{3}$$

but they differ in whether the history and residual are stored in CP2K's general tight-binding mixing infrastructure or inside TBLITE.

For linear-scaling self-consistent field calculations and the orbital- transformation route, the separate SCC-variable mixer is bypassed. In these cases CP2K optimizes the density

matrix itself and updates the GFN2-xTB variables directly from the current density,

$$\mathbf{x}^{(n)} = \mathbf{x}[P^{(n)}], \qquad H^{(n)} = H^0 + \Delta H[\mathbf{x}^{(n)}]. \tag{4}$$

This avoids mixing two electronic optimization problems at once. The present orbital-transformation implementation is restricted to Γ-point calculations without electronic smearing or additional virtual orbitals, while the diagonalization route supports the k-point machinery summarized in the main text. For diagonalization calculations with fractional occupations, the free-energy term can be interpreted in the finite-temperature framework of Mermin for Fermi-Dirac occupations, while Gaussian and related broadened occupations are part of the standard smearing machinery for Brillouin-zone integrations.[5–7]

2 K-Point Sampling Details

The main text gives the Bloch transformation and Brillouin-zone reconstruction used for periodic GFN2-xTB. At the input level, CP2K supports the standard Monkhorst-Pack grids and the MacDonald shifted meshes, as well as explicitly supplied closed k-point sets, for the diagonalization route.[8,9] For even Monkhorst-Pack meshes, Nyquist images require special bookkeeping so that symmetry-equivalent boundary images are not counted twice. When atomic symmetry is enabled, symmetry-equivalent k points can be reduced either by CP2K's established K290 machinery or by the SPGLIB backend, which applies the symmetry operations returned by the crystal-symmetry library, including fractional translations.[10] In practice, this makes the periodic GFN2-xTB implementation compatible with the same irreducible-k-point machinery used by CP2K for conventional periodic electronic structure calculations. All production benchmarks reported here use full symmetry reduction. The overlap-covariant Bloch phases include the fractional translations associated with nonsymmorphic crystal operations, so energies, analytical forces, and analytical stresses are invariant with respect to the corresponding unreduced meshes.

3 Stress and Virial Convention

The stress tensor reported to CP2K is treated as the derivative of the periodic total energy with respect to a homogeneous cell deformation. In the present implementation the TBLITE stress contribution is accumulated into CP2K's virial convention before it is combined with the remaining CP2K stress components.

4 Numerical Validation of Forces and Stress

The analytical periodic derivatives were validated independently in the native TBLITE CLI and in CP2K. Standalone CLI finite-difference tests gave maximum force-component and virial-component residuals of 2.19×10^{-8} and 1.11×10^{-7} a.u. for GFN1-xTB and 6.36×10^{-9} and 4.93×10^{-8} a.u. for GFN2-xTB, respectively. Representative CP2K native-Bloch calculations with full symmetry reduction gave summed absolute finite-difference residuals no larger than 7.7×10^{-7} a.u. for forces and 2.39×10^{-7} a.u. for stress, corresponding to relative deviations below 0.01%. As a separate end-to-end check of the common Γ path, CP2K-native and CLI calculations on all 23 final Γ-optimized X23b structures per method agree to maximum energy differences of 1.34×10^{-8} and 1.01×10^{-8} hartree, maximum force-component differences of 2.55×10^{-7} and 8.95×10^{-7} a.u., and maximum virial-component differences of 1.35×10^{-6} and 4.45×10^{-6} a.u. for GFN1-xTB and GFN2-xTB, respectively.

5 Scope of the GFN1-xTB–GFN2-xTB Comparison

The benchmark pairs in the main text compare the complete published GFN1-xTB and GFN2-xTB parametrizations. Beyond anisotropic electrostatics, the two models differ in several coupled Hamiltonian, repulsion, and dispersion terms. In GFN2-xTB, the multipolar variables enter the self-consistent Hamiltonian, while the charge-dependent D4 dispersion contribution responds to the same electronic state. Suppressing the multipolar contribu-

tion while retaining the remaining published parameters would therefore define an unparameterized hybrid rather than a recognized GFN2-xTB variant and would not isolate one additive energy term. We consequently restrict all accuracy claims to the complete GFN1-xTB and GFN2-xTB models. The implementation claim is tested independently by the finite-difference and CP2K–CLI consistency checks reported above.

6 DMC-ICE13 Relative-Energy Benchmark Data

Table S1 reports the k-point-dependent aggregate error statistics. Table S2 then reports the phase-resolved DMC-ICE13 relative energies and the corresponding GFN-xTB errors on the converged 3^3 mesh. The main-text values use native Bloch k-point sampling with full symmetry reduction and the Γ-centered 3^3 mesh employed for the published GGA, meta-GGA, and vdW-inclusive single-point DFT calculations in Ref. 11; the denser 4^3 and 5^3 meshes document residual Brillouin-zone convergence.

Table S1: K-point dependence of the aggregate DMC-ICE13 relative-energy errors in kJ mol^{-1} per water molecule. All errors are evaluated with respect to ice Ih over the twelve non-reference polymorphs. The explicit 2^3 mesh is already close to the converged aggregate statistics, and the 3^3, 4^3, and 5^3 Γ-centered meshes are converged on the scale of the benchmark errors.

Mesh	Method	ME	MAE	RMSE	MaxAE
Γ	GFN1-xTB	-5.29	6.69	7.15	10.47
Γ	GFN2-xTB	0.91	5.58	10.19	33.46
2^3	GFN1-xTB	-7.96	7.96	8.60	13.59
2^3	GFN2-xTB	-2.78	3.51	3.86	6.35
3^3	GFN1-xTB	-8.01	8.01	8.65	13.66
3^3	GFN2-xTB	-2.84	3.46	3.81	6.35
4^3	GFN1-xTB	-8.01	8.01	8.65	13.66
4^3	GFN2-xTB	-2.84	3.46	3.81	6.35
5^3	GFN1-xTB	-8.01	8.01	8.65	13.66
5^3	GFN2-xTB	-2.84	3.46	3.81	6.35

Table S2: DMC-ICE13 relative energies in kJ mol^{-1} per water molecule with respect to ice Ih using a Γ-centered 3^3 k-point mesh. The GFN1-xTB and GFN2-xTB values are periodic CP2K/TBLITE single-point calculations on the DMC-ICE13 reference geometries; errors are given relative to the DMC reference values of Ref. 11.

		GFN1-xTB		GFN2-xTB	
Phase	DMC	ΔE	Error	ΔE	Error
Ih	0.00	0.00	0.00	0.00	0.00
II	0.31	-7.56	-7.87	-3.41	-3.72
III	1.25	-3.60	-4.85	-1.99	-3.24
IV	3.83	-3.82	-7.65	-0.64	-4.47
VI	1.78	-10.14	-11.92	-2.88	-4.66
VII	4.99	-3.92	-8.91	8.40	3.41
VIII	4.23	-9.43	-13.66	4.57	0.34
IX	0.60	-5.02	-5.62	-2.69	-3.29
XI	0.16	-1.36	-1.52	-0.43	-0.59
XIII	2.12	-6.27	-8.39	-4.23	-6.35
XIV	1.70	-7.07	-8.77	-1.24	-2.94
XV	1.74	-10.02	-11.76	-2.71	-4.45
XVII	1.75	-3.40	-5.15	-2.37	-4.12

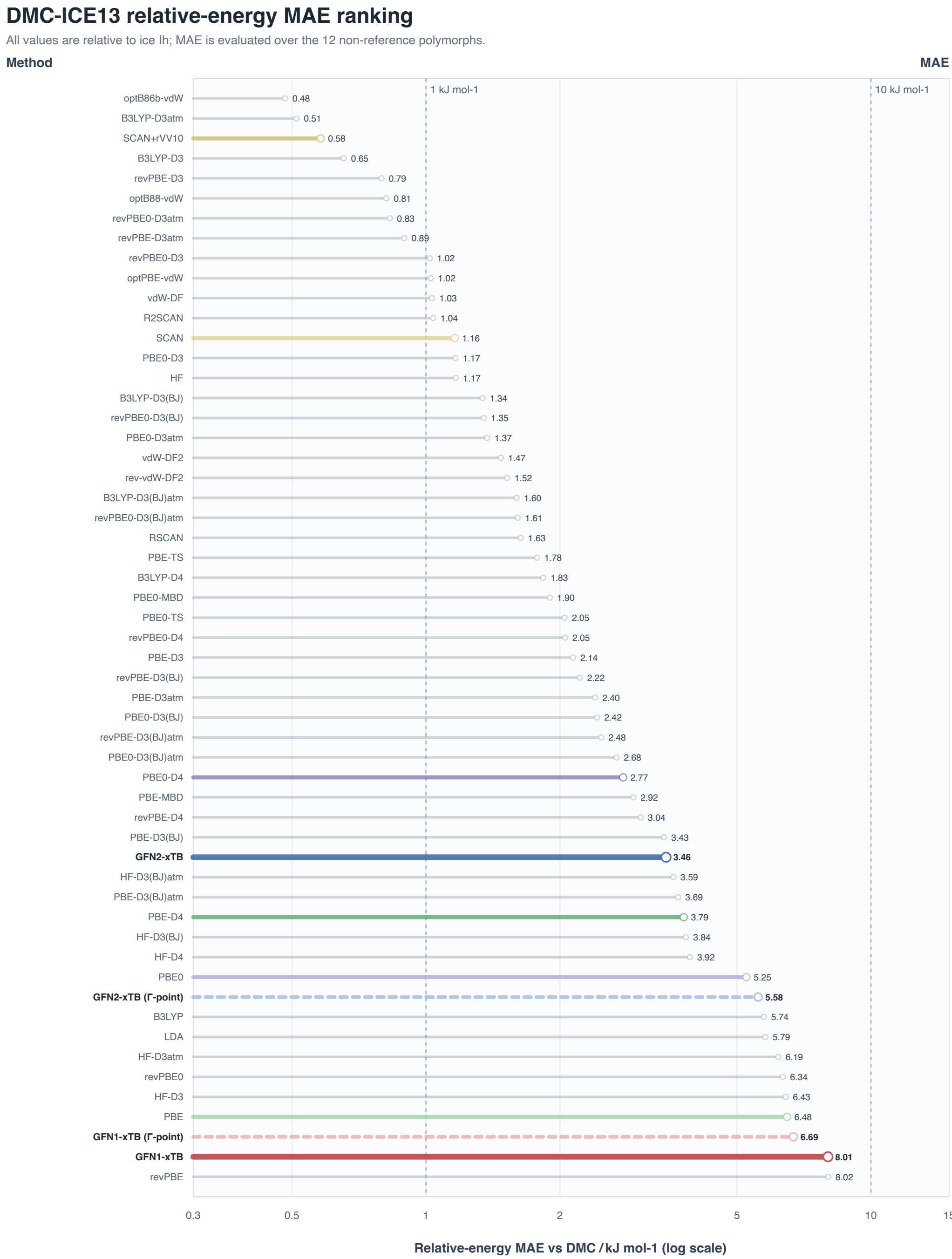

Figure S1: Relative-energy MAE ranking for periodic GFN1-xTB and GFN2-xTB and published DFT data from Della Pia *et al.*, J. Chem. Phys. **157**, 134701 (2022);[11] the GFN entries are CP2K/TBLITE single-point calculations from this work. The primary GFN entries use the Γ-centered 3^3 mesh; pale dashed entries show the Γ-point-only error compensation. Colored DFT entries correspond to the DFT curves shown in the main-text error plot. All methods are ranked by MAE over the twelve non-reference polymorphs; the horizontal axis is logarithmic.

7 LC10 Equation-of-State Benchmark Data

Table S3 reports the ten system-resolved native-Bloch 4^3 equation-of-state results. Conventional cubic eight-atom cells were used throughout, and cohesive energies employ isolated-atom CP2K/TBLITE references with the appropriate atomic spin states. Final single-point checks on 3^3, 4^3, and 5^3 meshes show that the cohesive energies are converged at the scale relevant to the aggregate errors. This compact main-group set tests covalent and ionic periodic bonding but does not cover transition-metal oxides, mixed-valence compounds, or multicomponent solids with more difficult charge-transfer behavior.

Table S3: LC10 lattice constants a in Å and cohesive energies E_{coh} in eV per atom. Experimental values include the zero-point correction used by Goldzak et al.[12]

Solid	a_{exp}	a_{GFN1}	a_{GFN2}	$E_{\mathrm{coh}}^{\mathrm{exp}}$	$E_{\mathrm{coh}}^{\mathrm{GFN1}}$	$E_{\mathrm{coh}}^{\mathrm{GFN2}}$
C	3.553	3.5585	3.5549	7.55	10.783	10.100
Si	5.421	5.6187	5.4462	4.70	5.933	3.738
SiC	4.347	4.5234	4.4127	6.47	8.353	6.817
BN	3.593	3.6252	3.6302	6.76	8.297	9.539
BP	4.525	4.5785	4.5501	5.14	6.196	6.225
AlN	4.368	4.6121	4.3128	5.85	7.623	8.400
AlP	5.448	5.5741	5.5450	4.31	6.865	5.009
MgS	5.188	5.0625	5.1442	4.04	3.657	4.604
LiF	3.973	3.7457	4.0040	4.46	5.215	4.887
LiCl	5.072	4.8091	4.8301	3.58	4.611	4.611

8 X23b Supporting Benchmark Data

The X23b k-point convergence test was performed as a fixed-geometry diagnostic on the reference crystal structures. It is therefore distinct from the final X23b relaxed-cell statistics in the main text, which use native Bloch 2^3-mesh crystal cell optimizations with full symmetry reduction and optimized gas-phase monomer references. For the fixed-geometry single-point energies, the aggregate errors are essentially converged between the 2^3 and 3^3 native Bloch meshes with full symmetry reduction. The remaining very large single-point errors are dominated by the sensitivity of the transferable xTB Hamiltonian to unrelaxed

molecular-crystal geometries and should not be interpreted as final cohesive-energy errors. A second k-point test was therefore performed on the fully relaxed native-Bloch 2^3 cells used in the main-text benchmark. The mean absolute lattice-energy changes from the 2^3 optimization mesh to a 3^3 single-point mesh are 1.192 and 1.120 kJ mol^{-1} for GFN1-xTB and GFN2-xTB, respectively. Increasing the single-point mesh from 3^3 to 4^3 reduces these changes to 0.079 and 0.084 kJ mol^{-1}, establishing the convergence of the 3^3 energies reported in the main text without replacing the underlying k-point-sampled cell optimizations. The X23b reference-value uncertainty estimated by Dolgonos, Hoja, and Boese from the spread of the vibrational back-corrections is much smaller than the dominant GFN-xTB outliers, with a mean $\Delta_{\max}$ of 0.93 kJ mol^{-1} and a maximum value of 2.0 kJ mol^{-1} over the 23 systems.[13]

Table S4: K-point dependence of fixed-geometry X23b single-point lattice-energy errors in kJ mol^{-1} per molecule. Errors are evaluated relative to the X23b reference lattice energies over all 23 molecular crystals. The single-point entries were evaluated on the reference crystal structures with Γ, 2^3, and 3^3 k-point meshes. The aggregate fixed-geometry errors are essentially converged between the 2^3 and 3^3 meshes. The corresponding relaxed-cell benchmark is reported in the main text. The lower block reports energy convergence on the final 2^3-optimized cells; the 3^3 rows provide the manuscript lattice-energy statistics and the 4^3 rows are the convergence check.

Mesh	Method	ME	MAE	RMSE	MaxAE
Γ	GFN1-xTB	-133.82	136.81	204.15	667.34
Γ	GFN2-xTB	-135.57	135.86	203.50	667.73
1^3	GFN1-xTB	-133.82	136.81	204.15	667.34
1^3	GFN2-xTB	-135.57	135.86	203.50	667.73
2^3	GFN1-xTB	-123.51	123.78	190.31	647.89
2^3	GFN2-xTB	-124.32	124.33	187.27	642.90
3^3	GFN1-xTB	-123.40	123.67	190.24	647.87
3^3	GFN2-xTB	-124.17	124.26	187.18	642.85
Final native-Bloch 2^3-optimized cells					
2^3	GFN1-xTB	-0.93	11.34	14.62	36.56
2^3	GFN2-xTB	-13.13	15.07	23.28	86.82
3^3	GFN1-xTB	0.26	11.35	14.02	30.94
3^3	GFN2-xTB	-12.02	14.09	21.34	77.79
4^3	GFN1-xTB	0.18	11.37	14.08	31.58
4^3	GFN2-xTB	-12.10	14.17	21.58	79.19

Figure S2 summarizes the full distribution of system-resolved X23b lattice-energy deviations. The DMC-X23 and multi-level coupled-cluster data are included as independent

high-accuracy scales for the molecular-crystal family, whereas the GFN1-xTB and GFN2-xTB rows are the native Bloch 3^3 energy evaluations on the 2^3-mesh relaxed cells discussed in the main text. The corresponding aggregate statistics are reported in Table 2 of the main text.

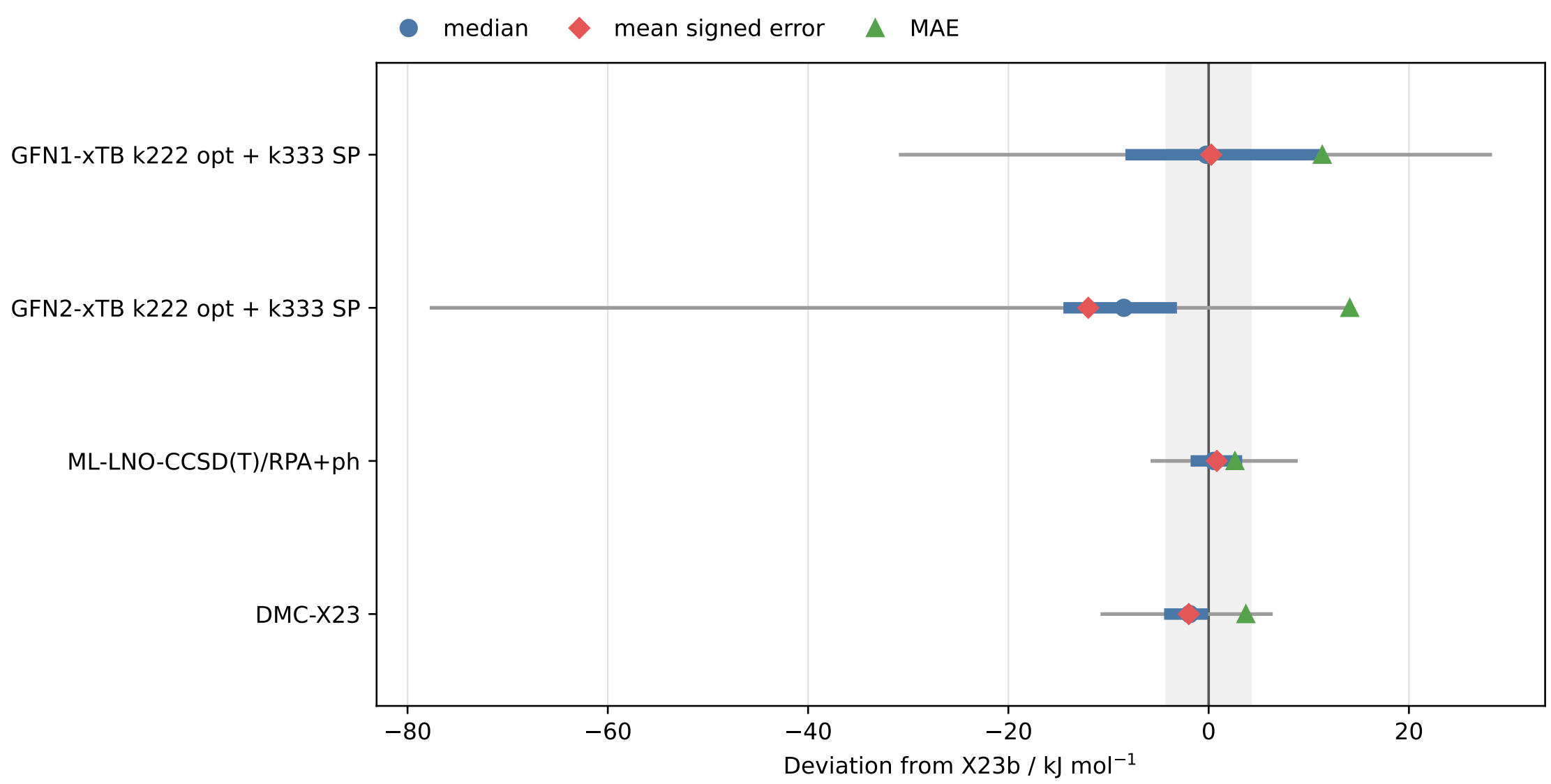

Figure S2: Distribution of system-resolved X23b lattice-energy deviations using 3^3 energies on the final 2^3-optimized cells. Grey whiskers indicate the minimum–maximum range over the 23 molecular crystals, the thick blue bars indicate the interquartile range, the blue circle marks the median, the red square marks the mean signed error, and the green triangle marks the mean absolute error. The grey band corresponds to ± 4.184 kJ mol^{-1} per molecule. The DMC-X23 and ML-LNO-CCSD(T)/RPA+ph data are taken from Refs. 14 and 15, respectively, and serve as independent high-accuracy reference scales.

For a directly comparable DFT context, we also evaluated the electronic DFT+D3 cell volumes reported by Dolgonos, Hoja, and Boese for the same X23b systems.[13] This comparison is limited to cell volumes, because the X23b reference volumes are explicitly back-corrected to the minimum of the electronic energy. The corresponding DFT+D3 energetic components are not used as an independent lattice-energy benchmark here, since they enter the construction of the X23b reference values.

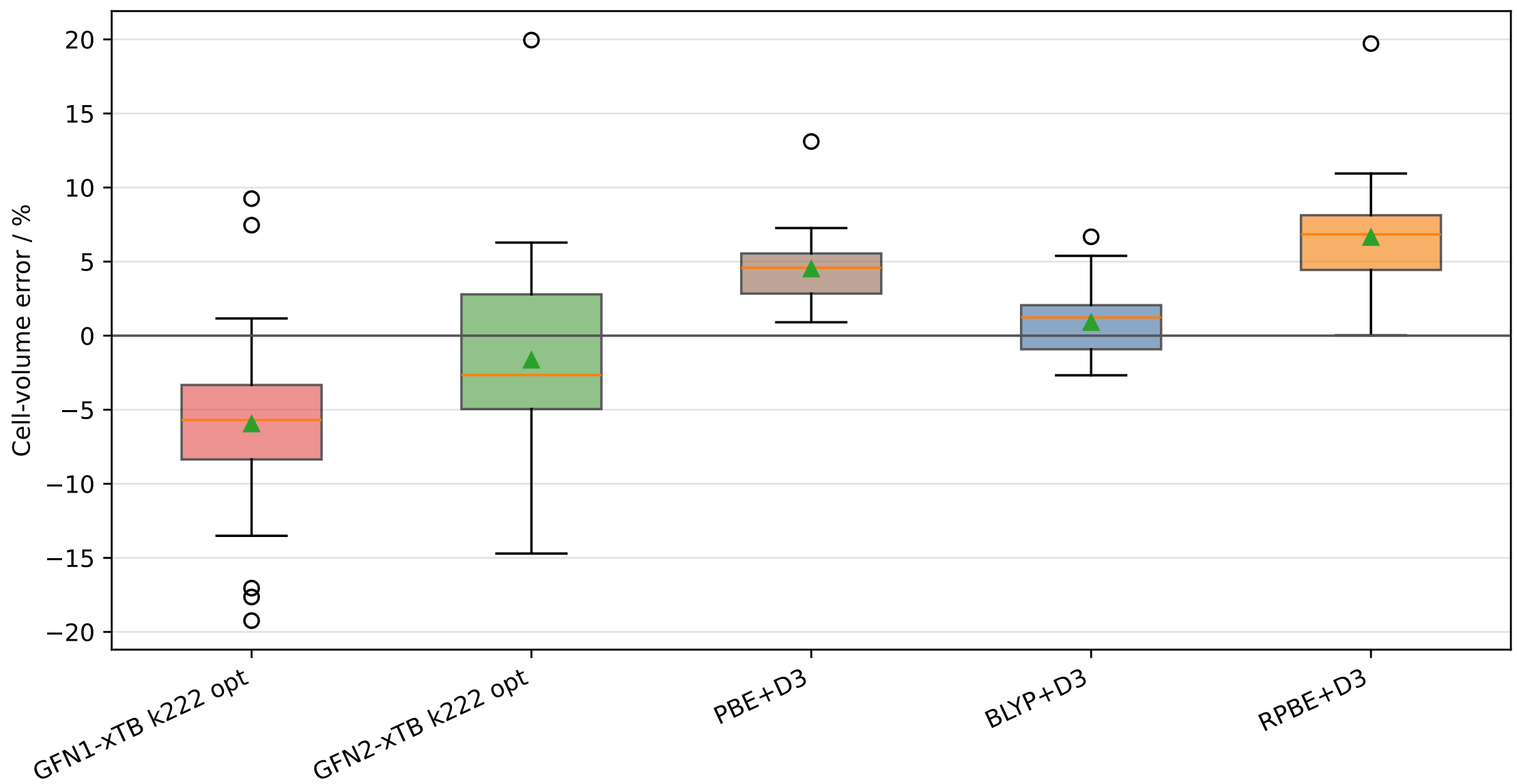

Figure S3: Distribution of relative X23b cell-volume errors for the present native Bloch 2^3-mesh relaxed GFN1-xTB and GFN2-xTB structures and for the PBE+D3, BLYP+D3, and RPBE+D3 electronic volumes reported by Dolgonos, Hoja, and Boese.[13] Grey whiskers indicate the minimum–maximum range over all 23 molecular crystals, the thick blue bars indicate the interquartile range, the blue circle marks the median, the red square marks the mean signed error, and the green triangle marks the mean absolute error. The grey band denotes ±5%, and the thin horizontal separator distinguishes the present GFN-xTB calculations from the DFT+D3 reference-volume data.

References

(1) Broyden, C. G. A Class of Methods for Solving Nonlinear Simultaneous Equations. *Math. Comput.* **1965**, *19*, 577–593.

(2) Johnson, D. D. Modified Broyden's Method for Accelerating Convergence in Self-Consistent Calculations. *Phys. Rev. B* **1988**, *38*, 12807–12813.

(3) Pulay, P. Convergence Acceleration of Iterative Sequences. The Case of SCF Iteration. *Chem. Phys. Lett.* **1980**, *73*, 393–398.

(4) Pulay, P. Improved SCF Convergence Acceleration. *J. Comput. Chem.* **1982**, *3*, 556–560.

(5) Mermin, N. D. Thermal Properties of the Inhomogeneous Electron Gas. *Phys. Rev.* **1965**, *137*, A1441–A1443.

(6) Methfessel, M.; Paxton, A. T. High-Precision Sampling for Brillouin-Zone Integration in Metals. *Phys. Rev. B* **1989**, *40*, 3616–3621.

(7) Marzari, N.; Vanderbilt, D.; De Vita, A.; Payne, M. C. Thermal Contraction and Disordering of the Al(110) Surface. *Phys. Rev. Lett.* **1999**, *82*, 3296–3299.

(8) Monkhorst, H. J.; Pack, J. D. Special Points for Brillouin-Zone Integrations. *Phys. Rev. B* **1976**, *13*, 5188–5192.

(9) MacDonald, A. H. Comment on Special Points for Brillouin-Zone Integrations. *Phys. Rev. B* **1978**, *18*, 5897–5899.

(10) Togo, A.; Shinohara, K.; Tanaka, I. Spglib: A Software Library for Crystal Symmetry Search. *Sci. Technol. Adv. Mater. Methods* **2024**, *4*, 2384822.

(11) Della Pia, F.; Zen, A.; Alfè, D.; Michaelides, A. DMC-ICE13: Ambient and High Pressure Polymorphs of Ice from Diffusion Monte Carlo and Density Functional Theory. *J. Chem. Phys.* **2022**, *157*, 134701.

(12) Goldzak, T.; Wang, X.; Ye, H.-Z.; Berkelbach, T. C. Accurate thermochemistry of covalent and ionic solids from spin-component-scaled MP2. *J. Chem. Phys.* **2022**, *157*, 174112.

(13) Dolgonos, G. A.; Hoja, J.; Boese, A. D. Revised Values for the X23 Benchmark Set of Molecular Crystals. *Phys. Chem. Chem. Phys.* **2019**, *21*, 24333–24344.

(14) Della Pia, F.; Zen, A.; Alfè, D.; Michaelides, A. How Accurate Are Simulations and Experiments for the Lattice Energies of Molecular Crystals? *Phys. Rev. Lett.* **2024**, *133*, 046401.

(15) Syty, K.; Czekało, G.; Pham, K. N.; Modrzejewski, M. Multi-Level Coupled-Cluster Description of Crystal Lattice Energies. *J. Chem. Theory Comput.* **2025**, *21*, 5533–5544.
